# Survival outcomes and prognostic indicators in canine pancreatitis: A retrospective cohort study of acute kidney injury and concurrent diseases

**DOI:** 10.14202/vetworld.2025.2969-2980

**Published:** 2025-10-08

**Authors:** Weerapat Chawanlawuthi, Walasinee Sakcamduang, Sataporn Phochantachinda, Duangthip Chatchaisak

**Affiliations:** Department of Clinical Science and Public Health, Faculty of Veterinary Science, Mahidol University, Nakhon Pathom, Thailand

**Keywords:** acute kidney injury, blood urea nitrogen-to-creatinine ratio, canine pancreatitis, concurrent diseases, hematocrit, prognostic markers, survival analysis

## Abstract

**Background and Aim:**

Canine pancreatitis is often complicated by acute kidney injury (AKI) and systemic comorbidities, both of which may worsen clinical outcomes. This study aimed to evaluate survival rates in dogs with pancreatitis, stratified by the presence of AKI and other concurrent diseases, and to identify prognostic indicators for mortality.

**Materials and Methods:**

A retrospective cohort study was conducted at Prasu Arthorn Veterinary Teaching Hospital, Mahidol University, Thailand, from February 2021 to February 2023. Medical records of 146 dogs diagnosed with pancreatitis (serum canine pancreatic lipase ≥400 μg/L and clinical signs) were reviewed. Dogs were categorized into four groups: Pancreatitis alone (n = 24), pancreatitis with AKI (n = 28), pancreatitis with concurrent diseases (n = 57), and pancreatitis with both AKI and concurrent diseases (n = 34). Survival was analyzed using Kaplan–Meier curves and log-rank tests, while prognostic factors were assessed using Cox proportional hazards regression.

**Results:**

The overall mortality rate was 39.72% (58/146), with the highest mortality in dogs with AKI (Groups 2 and 4). Median survival was 4 days (95% confidence interval [CI]: 0.0–11.7) in Group 2 and 7 days (95% CI: 2.7–11.2) in Group 4, while median survival was not reached in Groups 1 and 3 due to high survival. Hematocrit (HCT) and blood urea nitrogen-to-creatinine ratio (BCR) were identified as independent predictors of mortality. Lower HCT (Hazard ratio [HR] = 0.967, 95% CI: 0.941–0.994, p = 0.019) and higher BCR (HR = 1.024, 95% CI: 1.007–1.041, p = 0.006) were significantly associated with increased risk of death.

**Conclusion:**

AKI is a major negative prognostic factor in canine pancreatitis, markedly reducing survival irrespective of concurrent systemic diseases. Readily available markers, such as HCT and BCR, provide practical tools for early triage and prognostic stratification. Incorporating these parameters into clinical decision-making may enhance outcomes by guiding intensive monitoring and targeted interventions.

## INTRODUCTION

Canine pancreatitis is a common clinical condition characterized by pancreatic inflammation. Chronic pancreatitis, often diagnosed postmortem through histopathological examination, affects approximately 34% of dogs, with higher prevalence in overweight and female animals [1]. Early diagnosis remains challenging, emphasizing the need for reliable diagnostic tools. Although pancreatic biopsy is the gold standard [2], less invasive methods such as fine-needle aspiration with cytology are frequently employed due to anesthesia-related risks [3]. Serum biomarkers, particularly canine pancreatic lipase (cPL), have become critical in diagnosis, with concentrations ≥400 μg/L considered diagnostic when consistent clinical signs are present [2]. Despite standard treatment protocols [4], the survival rate in acute pancreatitis averages 72.9% [5], but outcomes vary significantly with disease severity. Concurrent conditions, particularly acute kidney injury (AKI), markedly worsen prognosis, with mortality rates reaching 70.6% in dogs affected by both acute pancreatitis and AKI [6]. Pancreatitis can also induce myocardial injury, evidenced by elevated troponin I levels in dogs with high Spec cPL values [7]. Unlike previous studies that evaluated pancreatitis or AKI separately, the present study provides an integrated analysis of survival outcomes across four distinct clinical categories: Pancreatitis alone, pancreatitis with AKI, pancreatitis with concurrent disease, and pancreatitis with both AKI and concurrent disease.

Although pancreatitis in dogs is widely recognized as a life-threatening condition, most previous studies have evaluated either pancreatitis or AKI in isolation, without accounting for the complex interplay of concurrent systemic diseases. Mortality in pancreatitis has been variably reported, and while AKI has consistently emerged as a poor prognostic factor, little is known about how additional comorbidities alter survival outcomes when layered onto pancreatitis with or without AKI. Furthermore, prognostic indices in veterinary medicine have predominantly focused on inflammatory markers, such as neutrophil-to-lymphocyte ratio or acute-phase proteins, but these parameters may not fully capture the multifactorial progression of the disease. The role of routinely accessible hematologic and biochemical markers, such as hematocrit (HCT) and the blood urea nitrogen-to-creatinine ratio (BCR), has been underexplored, despite their potential utility in early triage and clinical decision-making. Importantly, there is a lack of large-scale, region-specific studies in Southeast Asia addressing survival outcomes in canine pancreatitis across different clinical contexts. This limits the generalizability of current knowledge and constrains veterinarians’ ability to apply evidence-based prognostic tools in diverse clinical settings.

The present study aimed to retrospectively evaluate survival outcomes in dogs diagnosed with pancreatitis, stratified into four clinical groups: pancreatitis alone, pancreatitis with AKI, pancreatitis with concurrent systemic diseases, and pancreatitis with both AKI and concurrent diseases. In addition, this study sought to identify independent prognostic markers associated with mortality, with particular emphasis on HCT and the BCR. By employing survival analysis and multivariate regression modeling, the study intended to generate clinically relevant evidence to enhance prognostic assessment, guide monitoring strategies, and ultimately improve treatment outcomes in dogs with pancreatitis.

## MATERIALS AND METHODS

### Ethical approval

This retrospective study was reviewed by the Institutional Animal Care and Use Committee of the Faculty of Veterinary Science, Mahidol University, Thailand. Formal approval was waived due to the retrospective design. All procedures complied with institutional and national guidelines for the care and use of animals.

### Study period and location

The study was conducted from February 2021 to February 2023. This was a single-center retrospective cohort study conducted at Prasu-Arthorn Veterinary Teaching Hospital, Mahidol University, Nakhon Prathom, Thailand. Medical records of canine patients diagnosed with pancreatitis were reviewed. Clinical data were collected directly from the hospital records or, when necessary, supplemented through phone interviews with owners.

### Case inclusion and exclusion criteria

#### Inclusion criteria

- Presence of ≥2 clinical signs consistent with pancreatitis (vomiting, diarrhea, abdominal pain, lethargy, or anorexia) [8]
- Availability of sufficient medical records, including cPL results and clinical notes
- Serum cPL concentration ≥400 μg/L, measured using the Vcheck assay (Bionote V2400, Korea) [9]
- Documented survival outcome within 60 days
- Diagnosis and treatment were performed at the study center.

#### Exclusion criteria

- Diagnosis or treatment performed outside the study institution
- Absence of required clinical signs or cPL testing, or cPL levels <400 μg/L
- Incomplete survival outcome data
- Duplicate records, such as multiple entries for the same patient or hospitalization episode.

### Clinical grouping of patients

Dogs were categorized into four clinical groups based on the presence or absence of AKI and concurrent systemic diseases:

- Group 1: Pancreatitis alone
- Group 2: Pancreatitis with AKI
- Group 3: Pancreatitis with concurrent diseases
- Group 4: Pancreatitis with both AKI and concurrent diseases.

Concurrent diseases were defined as non-renal comorbidities confirmed clinically or diagnostically at the time of pancreatitis diagnosis.

### Confirmation of pancreatitis

The diagnosis of pancreatitis was based on the presence of compatible clinical signs (vomiting, diarrhea, abdominal pain, and lethargy) together with a serum cPL concentration of ≥400 μg/L. When available, abdominal ultrasonography was used to support the diagnosis [8]. Differential diagnoses, such as primary gastrointestinal disorders or secondary systemic involvement, were excluded based on clinical history, physical examination, and laboratory/imaging results.

### Diagnostic definitions of AKI and concurrent diseases

AKI was diagnosed according to the IRIS guidelines, confirmed by a ≥0.3 mg/dL increase in serum creatinine within 48 h and clinical signs of oliguria or anuria [9].

Concurrent diseases were defined as non-renal comorbidities identified at the time of pancreatitis diagnosis. Examples include:

- Cardiovascular: Myxomatous mitral valve disease (MMVD), staged through echocardiography according to ACVIM guidelines [10].
- Endocrine: Diabetes mellitus (blood glucose >200 mg/dL with clinical signs) and hyperadrenocorticism (positive low-dose dexamethasone suppression test) [11]
- Renal: Chronic kidney disease (CKD), staged according to IRIS guidelines [12].

### Management of pancreatitis

Standard treatment protocols included intravenous fluid therapy, analgesia, anti-emetic medications, and dietary management [13]. Additional therapies were tailored to concurrent diseases. Treatment variability across cases was acknowledged as a study limitation.

### Definition of survival

Survival outcomes were assessed at 60 days post-diagnosis [8]. Dogs that died within this period, regardless of cause, were categorized as non-survivors. Clinical and clinicopathological data were compared between survivors and non-survivors.

### Data collection and variables

#### Data extracted from medical records included:

- Demographics (age, sex, and breed)
- Clinical signs at presentation
- Hematologic and biochemical parameters (complete blood count [CBC], chemistry, electrolytes, blood gas, and urinalysis)
- Survival status at 60 days post-diagnosis.

Reference intervals for laboratory parameters were based on the in-house standards of the Prasu Arthorn Veterinary Teaching Hospital. Data were organized using Microsoft Excel 2016 (Microsoft Corp., Washington, USA).

### Statistical analysis

Survival time was defined from the date of diagnosis to the date of death. The cause of death could not be determined for all cases; therefore, survival analysis focused on the association between death and concurrent disease status.

Statistical analysis was performed using IBM Statistical Package for the Social Sciences Statistics for Windows, Version 27.0 (IBM Corp., Armonk, NY, USA). Methods included:

- Kaplan–Meier analysis with log-rank test for survival distribution comparison
- Kruskal–Wallis test for continuous variables (age, weight, CBC, biochemistry, electrolytes, blood gas, and urinalysis)
- Univariate Cox proportional hazards regression to identify potential predictors (p < 0.05)
- Multivariate Cox regression for significant variables from univariate analysis.

Proportional hazard assumptions were checked using Schoenfeld residuals and log(-log) plots. p < 0.05 was considered statistically significant. No corrections were applied for multiple comparisons due to the exploratory nature of the study. Although no formal power analysis was performed, the sample size was considered adequate based on previous retrospective study by Kuzi *et al*. [8] in canine pancreatitis.

## RESULTS

### Case enrollment and population characteristics

**Figure 1:**
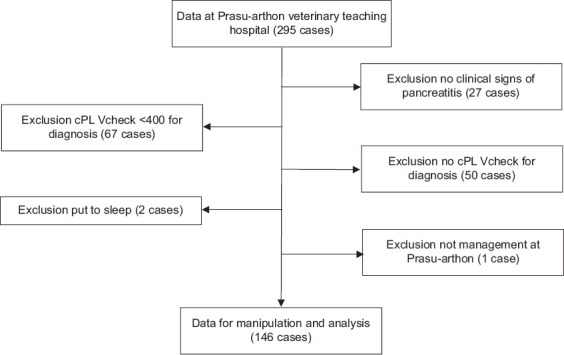
This figure illustrates the inclusion and exclusion criteria. A total of 146 cases were included in the analysis.

A total of 295 canine cases were initially reviewed. After excluding 149 cases based on predefined criteria (Figure 1), 146 dogs met the inclusion criteria and were enrolled in the study. The final cohort consisted of 73 males and 73 females, yielding a balanced sex distribution. Mixed-breed dogs represented nearly one-quarter of the study population (33/146, 22.6%). Among purebred dogs, the most common breeds were Pomeranians (31/146, 21.23%), Chihuahuas (21/146, 14.38%), Poodles (21/146, 14.38%), and Shih Tzus (14/146, 9.59%) (Table 1).

**Table 1:** Summary of the breeds in each group.

Breeds	n	Group 1	Group 2	Group 3	Group 4
Mixed	33 (22.6%)	1	10	14	8
Pomeranian	31 (21.23%)	10	3	14	4
Chi-Hua-Hua	21 (14.38%)	4	3	6	8
Poodle	21 (14.38%)	2	1	9	9
Shih-Tzu	14 (9.59%)	2	3	5	4
Yorkshire terrier	4	0	1	1	2
Jack Russell terrier	3	2	1	0	0
Siberian Husky	3	0	1	2	0
Pug	2	0	1	1	0
Thai Bangkaew	2	0	2	0	0
Golden retriever	2	1	0	1	0
Labrador retriever	1	0	0	1	0
Beagle	1	1	0	0	0
The West highland white terrier	1	0	0	1	0
Samoyed	1	0	0	0	1
American bully	1	0	1	0	0
Miniature bull terrier	1	0	1	0	0
Dachshund	1	0	0	1	0
Miniature Pinscher	1	0	0	1	0
Fila Brasileiro	1	1	0	0	0
Boxer	1	0	0	1	0
Total	146	24	28	58	36

Group 1 = Pancreatitis alone, Group 2 = Pancreatitis with acute kidney injury, Group 3 = Pancreatitis with concurrent disease, Group 4 = Pancreatitis with acute kidney injury and concurrent diseases

The most frequent clinical signs of pancreatitis were anorexia (81.51%), vomiting (80.13%), abdominal pain (69.17%), lethargy (45.89%), and diarrhea (42.46%).

### Clinical grouping of cases

The study population was stratified into four clinical groups:

- Group 1: Pancreatitis alone (n = 24).
- Group 2: Pancreatitis with AKI (n = 28).
- Group 3: Pancreatitis with concurrent diseases (n = 57).
- Group 4: Pancreatitis with both AKI and concurrent diseases (n = 34).

A summary of survival outcomes by group is presented in Table 2.

**Table 2:** Mortality rate in each patient group.

Group	Total n	Survivor	Death	Mortality rate (%)	Median survival time (day)	p-value
Group 1	23	22	1	4.34	-	
Group 2	28	10	18	64.28	4 (0.00–11.77)	<0.001
Group 3	58	44	14	31.81	-	
Group 4	36	11	25	69.44	7 (2.71–11.28)	<0.001
Total	146	87	58	39.72		

### Hematologic and biochemical findings

CBC and serum chemistry results are shown in Table 3. Non-survivors demonstrated significant abnormalities, including left-shift leukocytosis (p = 0.009) and azotemia (p < 0.001). In addition, several parameters were significantly lower in non-survivors: HCT (p = 0.009), red blood cell count (RBC) (p = 0.008), hemoglobin (Hb) (p = 0.011), total protein (TP) (p = 0.031), albumin (ALB) (p = 0.046), potassium (p = 0.045), and sodium-to-potassium ratio (p = 0.017).

**Table 3:** Comparison of variables in the death and alive groups.

Variable	n	Death	n	Alive	p-value
Body weight (kg)	57	6.20	89	7.30	0.729
Age (Year)	57	11.20	89	10.00	0.155
White blood cell count (10^3^/µL)	56	19.27	84	14.80	0.009*
Neutrophil (10^3^/µL)	55	15.56	83	11.55	0.004*
Lymphocyte (10^3^/µL)	55	3.47	83	2.58	0.061
Monocyte (10^3^/µL)	55	0.79	83	0.67	0.007*
Eosinophil (10^3^/µL)	55	0.00	83	0.00	0.768
N/L ratio	55	4.94	83	4.10	0.022*
P/L ratio	55	77.73	83	100.00	0.117
Hematocrit (%)	56	40.90	83	43.5	0.009*
Red blood cell (10^6^/µL)	57	59.47	83	78.07	0.008*
Hemoglobin (g/L)	57	59.95	83	77.75	0.011*
Mean corpuscular volume (fL)	57	68.98	83	71.54	0.714
Mean corpuscular hemoglobin level (pg)	57	70.52	83	70.49	0.997
Mean corpuscular Hb concentration (g/dL)	57	75.02	83	67.40	0.224
Platelet (10^3^/µL)	56	259.00	83	258.00	0.920
cPL	58	1402.50	88	1166.00	0.116
BUN (mg/dL)	54	99.00	76	26.00	<0.001*
Creatinine (mg/dL)	54	3.42	80	1.20	<0.001*
BUN/Creatinine ratio	54	25.19	78	16.33	0.002*
ALT (U/L)	50	93.50	79	67.00	0.041*
Alkaline phosphatase concentration (U/L)	49	437.00	74	248.00	0.051
Total protein (g/dL)	25	6.30	23	7.00	0.031*
Albumin (g/dL)	29	2.60	29	2.90	0.046*
Glucose (mg/dL)	22	95.00	25	99	0.343
pH	13	7.37	14	7.35	0.808
HCO_3−_ (mmol/L)	13	13.4	14	12.50	0.382
PCO_2_ (mmol/L)	13	4.01	14	3.37	1.000
Sodium (mmol/L)	35	139.70	43	143.40	0.213
Potassium (mmol/L)	34	3.97	43	3.60	0.045*
Chloride (mmol/L)	21	104.50	30	106.20	0.438
Sodium–potassium ratio	34	31.82	43	39.47	0.017*
Ionized calcium (mmol/L) concentration	9	1.03	11	1.09	0.648
Specific gravity of urine	19	1.02	27	1.018	0.545

N/L ratio = Neutrophil-to-lymphocyte ratio; P/L ratio = Platelet-to-lymphocyte ratio; cPL = Canine pancreatic lipase; BUN = Blood urea nitrogen; HCO_3−_ = Bicarbonate; PCO_2_ = Partial carbon dioxide. Data are reported as median (50^th^ quartile) with interquartile range (25^th^–75^th^ quartile).

* indicates statistical significance at p* <* 0.05

Significant differences (p < 0.05) were also identified across groups for age, WBC, neutrophils, monocytes, lymphocytes, HCT, RBC, Hb, blood urea nitrogen (BUN), creatinine, BCR, alanine transaminase (ALT), and alkaline phosphatase (ALP) (Table 4).

**Table 4:** Baseline characteristics of the dogs.

Variable	n	Value Group 1	n	Value Group 2	n	Value Group 3	n	Value Group 4	p-value
Breed pure/Mixed (%)	24	95.83/4.17	28	64.29/35.71	58	75.87/24.13	36	77.78/22.22	0.059
Gender M/F (%)	24	58.33/41.67	28	50/50	58	50/50	36	44.44/55.56	0.776
Age (Year)	24	5.00 (3.50–9.00)^c^	28	10.00 (5.65–13.30)^e^	58	10.00 (8.00–11.92)^f^	36	13.2 (10.02–15.15)^c,e,f^	0.002
WBC (10^3^/µL)	23	14.54 (11.24–22.21)^c^	26	17.22 (10.87–19.58)^e^	57	17.25 (11.42–24.99)^f^	35	21.17 (13.93–40.71)^c,e,f^	0.020
Neutrophil (10^3^/µL)	23	10.79 (8.20–17.51)^c^	26	13.42 (8.22–15.61)^e^	57	12.42 (8.12–19.28)^f^	33	17.86 (11.44–33.1)^c,e,f^	0.013
Lymphocyte (10^3^/µL)	23	2.67 (2.29–4.05)	26	2.40 (1.69–3.45)^e^	57	2.8 (1.99–3.89)^f^	33	3.96 (2.30–5.87)^e,f^	0.012
Monocyte (10^3^/µL)	23	0.7 (0.32–0.91)^c^	26	0.63 (0.46–1.02)^e^	57	0.71 (0.37–1.00)^f^	33	0.87 (0.62–1.69)^c,e,f^	0.031
Eosinophil (10^3^/µL)	23	0.0 (0.0–0.0)	26	0.00 (0.00–0.12)	57	0.00 (0.00–0.25)	33	0.00 (0.00–0.42)	0.093
N/L ratio	23	3.97 (3.23–4.53)	26	4.65 (3.73–6.26)	57	4.73 (3.51–5.57)	33	4.81 (3.85–6.62)	0.238
P/L ratio	23	95 (61.49–176.32)	26	105.76 (51.95–188.23)	57	78.45 (42.68–158.17)	33	78.96 (25.84–144.02)	0.433
Hematocrit (%)	23	49 (42.4–52.8)^b,c^	26	47.45 (41.32-55.17)^d,e^	57	42.8 (34.45–46.70)^b,d,f^	34	35.8 (30.32–41.30)^c,e,f^	<0.001
Red blood cell (10^6^/µL)	23	6.75 (6.19–7.29)^b,c^	27	6.93 (6.12–7.74)^d,e^	57	6.23 (5.02–6.48)^b,d,f^	33	5.26 (4.19–6.05)^c,e,f^	<0.001
Hemoglobin (g/L)	23	16.7 (14.20–17.80)^b,c^	27	16.2 (13.70–19.00)^d,e^	57	14.30 (11.65–15.85)^b,d,f^	33	12.80 (10.15–14.35)^c,e,f^	<0.001
Mean corpuscular volume (fL)	23	71.40 (68.20–74.70)	27	68.10 (66.20–72.40)	57	69.70 (66.90–72.05)	33	69.90 (67.40–73.05)	0.284
Mean corpuscular hemoglobin level (pg)	23	24.40 (23.20–25.30)	27	23.60 (22.80–24.80)	57	23.60 (22.50–24.55)	33	23.60 (23.00–24.55)	0.284
Mean corpuscular Hb concentration (g/dL)	23	34.00 (33.50–34.50)	27	34.10 (33.40–34.50)	57	33.90 (33.15–34.70)	33	33.80 (33.00–34.70)	0.702
Platelet (10^3^/µL)	23	268 (227–356)	26	261 (164–353)	57	231 (142–379)	34	275 (157–478)	0.412
BUN (mg/dL)	21	14.0 (11.5–21.5)^a,c^	27	100 (59.0–160.0)^a,d^	47	21 (11.0–30.0)^d,f^	36	95 (73–180)^c,f^	<0.001
Creatinine (mg/dL)	22	1.07 (0.98–1.20)^a,c^	27	4.85 (2.47–7.37)^a,d^	50	1.08 (0.93–1.44)^d,f^	36	3.07 (2.04–6.96)^c,f^	<0.001
BUN/creatinine ratio	22	13.91 (8.72–20.68)^a^	27	18.29 (14.28–26.08)^e^	47	15.72 (10.02–27.82)^f^	36	30.48 (23.95–45.43)^e,f^	<0.001
ALT (U/L)	22	42 (29–78)^b,c^	24	77 (30–139)	50	103 (39–289)^b^	34	81 (45–162)^c^	0.020
Alkaline phosphatase concentration (U/L)	21	182 (59.5–310.5)^b,c^	23	215 (86–616)^d^	47	495 (174–1128)^b,d^	33	394 (115–863)^c^	0.004
Total protein (g/dL)	4	6.50 (5.25–7.07)	12	6.70 (5.87–7.82)	20	6.7 (6.25–7.25)	13	6.2 (6.0–7.3)	0.793
Albumin (g/dL)	4	2.75 (2.37–3.20)	17	2.50 (2.25–3.00)	24	2.75 (2.50–3.00)	14	2.60 (2.37–2.72	0.515
Globulin (g/dL)	3	3.80	12	4.45 (3.57–4.72)	19	4.0 (3.3–4.5)	13	3.70 (3.45–4.15)	0.499
Globulin ratio	3	0.85	12	0.56 (0.53–0.80)	20	0.70 (0.53–0.88)	13	0.68 (0.63–0.83)	0.494
Glucose (mg/dL)	3	91	10	94.5 (81–102)	17	99 (86–128)	18	99 (70–465)	0.796
pH	1	7.36	5	7.26 (7.19–7.38)	11	7.34 (7.27–7.39)	10	7.39 (7.27–7.43)	0.352
HCO_3_− (mmol/L)	1	21.3	5	12.7 (8.75–16.65)	11	12.4 (9.4–19.6)	10	15.25 (12.25–24.95)	0.318
PCO_2_ (mmol/L)	1	5.00	5	3.05 (2.82–4.27)	11	3.22 (2.74–4.57)	10	4.02 (3.09–6.46)	0.423
Sodium (mmol/L)	10	144.45 (142.22– 147.72)	17	140.0 (130.7–144.9)	28	142.75 (137.72–145.22)	23	139 (134–149)	0.487
Potassium (mmol/L)	10	3.80 (3.39–4.13)	17	3.85 (3.29–4.89)	28	3.52 (2.98–4.22)	22	3.75 (3.27–4.83)	0.252
Chloride (mmol/L)	9	108.90 (105.05–110.45)	11	104.5 (89.9–111.5)	18	106.20 (100.15–110.00)	13	102.6 (98.05–107.75)	0.563
Sodium–potassium ratio	10	38.59 (35.00–42.74)	17	31.89 (29.13–43.55)	28	39.81 (33.72–45.08)	22	36.44 (29.33–43.08)	0.149
Ionized calcium (mmol/L) concentration	1	1.26	3	1.11	8	1.07 (1.01–1.13)	8	0.98 (0.69–1.15)	0.462
Specific gravity of urine	3	1.011	10	1.010 (1.000–1.020)	14	1.025 (1.015–1.036)	19	1.018 (1.013–1.020)	0.079

a Significant difference compared with pancreatitis (Group 1) and pancreatitis with AKI (Group 2) (p < 0.05).

b Significant difference compared with pancreatitis (Group 1) and pancreatitis with concurrent disease (Group 3) (p < 0.05).

c Significant difference compared with pancreatitis (Group 1) and pancreatitis with AKI and concurrent disease (Group 4) (p < 0.05).

d Significant difference compared with pancreatitis with AKI (Group 2) and pancreatitis with concurrent disease (Group 3) (p < 0.05).

e Statistically significant difference compared with pancreatitis with AKI (Group 2) and pancreatitis with AKI and concurrent disease (Group 4) (p < 0.05).

f Statistically significant difference compared with pancreatitis with concurrent disease (Group 3) and pancreatitis with AKI and concurrent disease (Group 4) (p < 0.05). Group 1 = Pancreatitis alone, Group 2 = Pancreatitis with acute kidney injury, Group 3 = Pancreatitis with concurrent disease, and Group 4 = Pancreatitis with acute kidney injury and concurrent diseases, N/L ratio = Neutrophil-to-lymphocyte ratio, P/L ratio = Platelet-to-lymphocyte ratio, cPL = Canine pancreatic lipase, BUN = Blood urea nitrogen, HCO_3−_ = Bicarbonate, PCO_2_ = Partial carbon dioxide. Data are reported as median (50^th^ quartile) with interquartile range (25^th^–75^th^ quartile)

- Inflammatory response: Group 4 exhibited the most severe inflammation, with the highest WBC (21.17 × 10^3^/μL), neutrophil (17.86 × 10^3^/μL), and monocytes counts (0.87 × 10^3^/μL)
- Erythrocyte indices: Group 4 also had the lowest HCT (35.8%), reflecting pronounced anemia, although platelet counts were not significantly different among groups
- Renal function: Groups 2 and 4 showed the most severe renal dysfunction, with markedly elevated BUN (100 and 95 mg/dL, respectively) and creatinine (4.85 and 3.07 mg/dL, respectively)
- Liver involvement: Group 3 demonstrated the highest liver enzyme activities (ALT: 103 U/L; ALP: 495 U/L), suggesting greater hepatic involvement. No significant group differences were observed for electrolyte or acid–base balance parameters.

### Concurrent diseases

MMVD was the most frequently associated comorbidity, particularly in Groups 3 and 4. In Group 3, the second most common comorbidity was diabetes mellitus (DM), whereas in Group 4, it was CKD. Hepatic involvement was the third most frequent condition in Group 3, while DM was the third most common in Group 4 (Table 5).

**Table 5:** Summary of concurrent systemic disease in Groups 3 and 4.

System	Concurrent disease	Group 3 (58) (%)	Group 4 (36) (%)
Urinary system	CKD	8 (13.79)	12 (33.33)
	Proteinuria without AKI or CKD	1 (1.72)	0
	Cystitis	0	0
Cardiovascular system	MMVD unstaged	1 (1.72)	4 (11.11)
	MMVD stage B1	10 (17.24)	8 (22.22)
	MMVD stage B2	2 (3.44)	2 (5.56)
	MMVD stage C	1 (1.72)	3 (8.33)
	MMVD stage D	1 (1.72)	1 (2.78)
Respiratory system	Lung involvement	3 (5.17)	0
	Tracheal collapse	0	1 (2.78)
Neuro-ophthalmic system	Brain involvement	5 (8.62)	3 (8.33)
	Spinal cord involvement	0	1 (2.78)
	Ophthalmic involvement	1 (1.72)	3 (8.33)
Endocrine system	Diabetes mellitus	7 (12.07)	5 (13.89)
	Diabetic ketoacidosis	4 (6.89)	3 (8.33)
	Hyperadrenocorticism	2 (3.44)	2 (5.56)
Gastrointestinal-hepatic system	Exocrine pancreatic insufficiency	1 (1.72)	0
	Gastro-intestinal involvement	3 (5.17)	1 (2.78)
	Hepatic involvement	11 (18.96)	0
Infectious and hematological systems	Infection with blood parasite	10 (17.24)	3 (8.33)
	Sepsis or SIRs	1 (1.72)	3 (8.33)
	Immune-mediated thrombocytopenia	1 (1.72)	0
	Immune-mediated hemolytic anemia	1 (1.72)	0
Reproductive system	Pyometra	1 (1.72)	2 (5.56)
Neoplasia	Lymphoma	3 (5.17)	1 (2.78)
	Mast cell tumor	2 (3.44)	0
	Hepatic mass	1 (1.72)	0
	Splenic mass	0	1 (2.78)
	Unidentified skin mass	0	1 (2.78)
	Prostatic tumor	1 (1.72)	0
	Epithelial carcinoma	1 (1.72)	0
	MGT	0	2 (5.56)
Emergency condition	Hypovolemic shock	1 (1.72)	1 (2.78)

MMVD = Myxomatous mitral valve disease, AKI = Acute kidney injury, CKD = Chronic kidney disease, SIRs = Systemic inflammatory response syndrome, MGT = Mammary gland tumor

### Survival analysis

**Figure 2:**
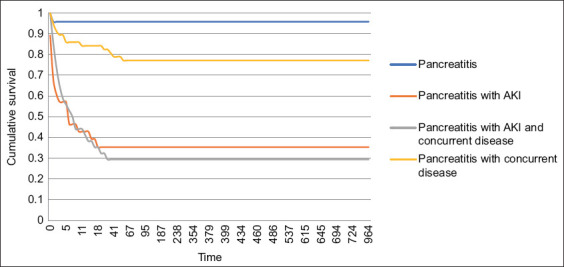
Kaplan–Meier survival analysis in each group. Group 1 (pancreatitis): n = 24, Group 2 (pancreatitis + acute kidney injury [AKI]): n = 28, median survival time (MST) 4 days, Group 3 (pancreatitis + concurrent disease): n = 57, and Group 4 (pancreatitis + AKI + concurrent disease): n = 34, MST 7 days.

Of the 146 dogs included in survival analysis, 58 (39.72%) died. Mortality was markedly higher in Groups 2 and 4 compared to Groups 1 and 3 (Figure 2).

- Group 2: Median survival 4 days (95% confidence interval [CI]: 0.0–11.7
- Group 4: Median survival 7 days (95% CI: 2.7–11.2)
- Groups 1 and 3: Median survival was not reached due to high survival rates.

No significant difference in survival was observed between Groups 2 and 4 (Table 2).

### Prognostic risk factors

Univariate Cox regression identified multiple predictors of mortality, including decreased HCT, RBC, Hb, and ALB, and increased WBC, neutrophil, BUN, creatinine, BCR, and potassium (Table 6).

**Table 6:** Univariate Cox proportional hazard analysis results.

Variable	HR	95% CI of the HR	p-value
Hematocrit	0.963	0.937–0.990	0.008
Red blood cell count	0.773	0.641–0.933	0.007
Hemoglobin	0.902	0.835–0.975	0.009
White blood cell count	1.007	1.001–1.012	0.021
Neutrophil	1.020	1.007–1.032	0.002
Monocyte	1.128	1.033–1.233	0.008
BUN level	1.009	1.006–1.012	<0.001
Creatinine	1.125	1.082–1.169	<0.001
BUN/Creatinine ratio	1.025	1.008–1.042	0.003
Total protein	0.629	0.425–0.931	0.024
Albumin	0.341	0.132–0.881	0.026
Potassium	1.498	1.034–2.169	0.033

HR = Hazard ratio, CI = Confidence interval, BUN = Blood urea nitrogen

Multivariate Cox regression confirmed two independent predictors:

- Lower HCT (Hazard ratio [HR] = 0.967, 95% CI: 0.941–0.994, p = 0.019)
- Higher BCR (HR = 1.024, 95% CI: 1.007–1.041, p = 0.006) (Table 7).

**Table 7:** Multivariate Cox proportional hazards analysis results.

Variable	HR	95% CI of the HR	p-value
BUN-to-creatinine ratio	1.024	1.007–1.041	0.006
Hematocrit	0.967	0.941–0.994	0.019

HR = Hazard ratio, CI = Confidence interval, BUN = Blood urea nitrogen

### Correlation analysis

Pearson’s correlation analysis revealed strong positive correlations among hematologic parameters:

- HCT, RBC, and Hb (r > 0.96, p < 0.01)
- WBC, neutrophils, and monocytes (WBC–neutrophils: r = 0.773; WBC–monocytes: r = 0.931; p < 0.01).

These clusters suggest integrated profiles of hematologic and inflammatory disruption in fatal cases.

Renal parameters also showed strong interdependence: BUN and creatinine were strongly correlated (r = 0.722, p < 0.01), while BUN moderately correlated with BCR (r = 0.449, p < 0.01). These findings highlight the combined contribution of renal compromise, anemia, and inflammation in determining survival outcomes (Supplementary data).

## DISCUSSION

### Prognostic significance of AKI and comorbidities

This study highlights the prognostic impact of AKI and comorbidities in canine pancreatitis and introduces a dual-marker approach using HCT and BCR. Findings demonstrate that inflammation, anemia, and organ dysfunction progressively worsen with increasing disease complexity, ultimately reducing survival. AKI emerged as the most critical determinant of outcome, with HCT and BCR identified as independent predictors of mortality. These results emphasize the importance of early recognition and monitoring of renal and hematologic parameters in improving outcomes. Unlike prior studies that dichotomized cases by AKI status, the four-group classification applied here provides a more nuanced understanding of layered clinical complexity.

### Mortality patterns across clinical groups

The overall mortality rate of 39.72% (58/146) was lower than previously reported but consistent with the high-risk profile of pancreatitis, particularly when complicated by AKI. Mortality was markedly higher in Group 2 (64.28%) and Group 4 (70.58%), confirming the poor prognosis associated with renal dysfunction. AKI is known to exacerbate systemic inflammation, drive multiorgan dysfunction, and shorten survival times, which was evident in the reduced median survival of Groups 2 and 4. Interestingly, the addition of non-renal comorbidities in Group 4 did not significantly worsen outcomes compared with AKI alone, suggesting that AKI itself is the dominant prognostic factor. In contrast, Group 3 (concurrent diseases without AKI) showed lower mortality, indicating that comorbidities alone did not markedly affect survival.

### Role of BUN, creatinine, and BCR

Univariate analysis identified BUN and creatinine as significant predictors of mortality. BUN reflects protein catabolism [14], gastrointestinal bleeding [15], and hepatic dysfunction [16], but its reliability is limited by extrarenal influences. Creatinine, a marker of glomerular filtration rate, is less affected by extrarenal factors [17, 18], but it rises more slowly in acute disease [19] and is influenced by muscle mass [20].

The BCR integrates information from both BUN and creatinine, making it a more comprehensive measure of renal function and systemic compromise. In this study, elevated BCR independently predicted mortality, with each unit increase raising the hazard of death by 2.4%. Elevated BCR has been associated with dehydration, hypovolemia [21], and gastrointestinal bleeding [22], all of which worsen pancreatitis outcomes. Thus, BCR provides additional clinical utility beyond individual markers, reflecting volume status, perfusion, and catabolic stress.

### Prognostic role of HCT

Reduced HCT was significantly associated with mortality, indicating the role of anemia in disease progression. Pancreatitis-related anemia may arise from gastrointestinal bleeding [4], chronic systemic inflammation altering iron metabolism and erythropoiesis [23], or immune-mediated hemolytic anemia [24]. The decrease in HCT likely reflects a chronic inflammatory state, consistent with anemia of critical illness.

Together, HCT and BCR provide accessible prognostic tools at presentation and during hospitalization. A combination of low HCT and rising BCR should prompt earlier evaluation of bleeding, impaired red blood cell production, or renal dysfunction. Early renal support, electrolyte correction [25], and renal replacement therapy [26] may improve survival in high-risk patients. This study is the first to validate HCT and BCR as independent prognostic markers using multivariate Cox regression in canine pancreatitis.

### Comparison with previous prognostic markers

A recent study identified red cell distribution width (RDW) and BUN as independent predictors of mortality in canine pancreatitis, with an overall survival rate of 72.9% [5]. In contrast, our study reported a lower survival rate (60.27%), likely due to the inclusion of patients with AKI and comorbidities. Importantly, BCR emerged as a stronger predictor than BUN alone, and HCT extended the prognostic spectrum beyond RDW. These differences underscore the importance of evaluating both conventional and derived markers across various clinical settings.

### Inflammatory markers and neutrophil count

Neutrophil count was also a significant univariate predictor of mortality, consistent with the role of systemic inflammation in pancreatitis. Neutrophilic activation contributes to cytokine release, tissue damage, and complications such as systemic inflammatory response syndrome and multiple organ dysfunction syndrome [27]. Previous research has linked elevated neutrophil-to-lymphocyte ratios with increased mortality and longer hospitalization in pancreatitis [28]. Although acute-phase proteins such as C-reactive protein (CRP) were not measured in this study, earlier studies have established their association with disease severity [29]. By identifying HCT and BCR as novel independent predictors, our findings extend the range of clinically accessible prognostic tools beyond traditional inflammatory indices.

### Concurrent diseases: Focus on MMVD

MMVD was the most frequently observed comorbidity, particularly in Groups 3 and 4, with Stage B1 MMVD being the most common. While MMVD B1 does not typically influence pancreatitis development or progression [10, 30], its high prevalence in affected dogs may reflect age-related physiological changes [31] or systemic inflammation as shared predisposing factors. The systematic mapping of concurrent diseases by organ system, as presented in Table 5, introduces a novel approach and may form the basis for a future pancreatitis comorbidity index (PCI) in veterinary medicine.

### Strengths and novel contributions

This study makes several important contributions to the literature on canine pancreatitis:

1. It represents one of the few large-scale retrospective analyses from Southeast Asia, adding region-specific data to global veterinary knowledge.
2. It introduces a four-tier classification framework that stratifies outcomes by AKI and comorbidity status, offering greater clinical precision than previous dichotomous models.
3. It is the first study to concurrently validate HCT and BCR as independent prognostic markers using multivariate Cox regression.
4. It applies correlation matrix analysis, revealing interdependent clusters of inflammatory and hematologic parameters, which enhances understanding of systemic pathophysiology.
5. It systematically maps comorbidities by organ system, potentially supporting the development of a veterinary PCI.

### Limitations

This study has several limitations inherent to its retrospective design. Treatment protocols were not standardized, and therapeutic approaches varied across cases, which may have influenced survival outcomes and limited the comparability of results. Important clinical information, including nutritional status, medication history, CRP levels, advanced imaging findings, and longitudinal follow-up data, was inconsistently available, restricting the comprehensiveness of the analysis. Concurrent diseases may also have been underreported or misclassified due to variability in diagnostic workups and clinician-dependent criteria, which can introduce potential bias.

In addition, the duration of hospitalization and the effects of specific treatment modalities on prognosis were not assessed, which further limited interpretation. Finally, causes of death could not be confirmed in all cases, while progression to systemic complications, such as suspected multiorgan dysfunction, was frequently noted; the absence of postmortem confirmation restricts certainty in attributing mortality.

## CONCLUSION

This retrospective study provides new insights into the prognostic landscape of canine pancreatitis by demonstrating the critical impact of AKI and systemic comorbidities on survival. Among 146 dogs included, the overall mortality was 39.72%, with the highest rates observed in dogs with AKI, either alone (64.28%) or in combination with concurrent diseases (70.58%). Kaplan–Meier analysis confirmed significantly shorter survival times in these groups, underscoring AKI as the dominant negative prognostic factor. Multivariate Cox regression further identified two readily accessible markers, HCT and the BCR, as independent predictors of mortality. Together, these findings highlight the interplay of renal dysfunction, anemia, and systemic inflammation as key determinants of outcome in canine pancreatitis.

From a clinical perspective, the identification of HCT and BCR as independent prognostic indicators offers practical tools for early triage, continuous monitoring, and guiding treatment intensity in affected dogs. Their availability in routine diagnostics allows clinicians to stratify patients at admission and prioritize intensive interventions for those at the highest risk. The group-specific survival patterns reported here may also support decision-making in critical care settings, particularly in prognostic discussions with owners and in the allocation of ICU resources.

Future studies should prospectively validate these findings using standardized treatment protocols, incorporating acute-phase proteins and imaging data, and conducting systematic follow-up to confirm the causes of death. The development of a pancreatitis comorbidity index (PCI) based on organ-specific mapping of concurrent diseases could further refine prognostic stratification and clinical decision-making.

This study reinforces AKI as the primary determinant of a poor prognosis in canine pancreatitis and introduces HCT and BCR as robust and accessible prognostic markers. Integrating these parameters into clinical practice may enhance risk assessment, optimize treatment strategies, and ultimately improve survival outcomes in affected dogs.

## DATA AVAILABILITY

The supplementary data can be made available from the corresponding author on request.

## AUTHORS’ CONTRIBUTIONS

WC, WS, SP, and DC: Conception and study design, data collection and analysis, clinical assessment and interpretations of the results. WC: Drafted the manuscript. WS, SP, and DC: Critical revision of the manuscript for important intellectual content. All authors have read and approved the final version of the manuscript.

## References

[1] Watson P.J, Roulois A.J.A, Scase T, Johnston P.E.J, Thompson H, Herrtage M.E (2007). Prevalence and breed distribution of chronic pancreatitis at post-mortem examination in first-opinion dogs. J. Small Anim. Pract.

[2] Xenoulis P.G (2015). Diagnosis of pancreatitis in dogs and cats. J. Small Anim. Pract.

[3] Cordner A.P, Sharkey L.C, Armstrong P.J, McAteer K.D (2015). Cytologic findings and diagnostic yield in 92 dogs undergoing fine-needle aspiration of the pancreas. J. Vet. Diagn. Invest.

[4] Lim S.Y, Cridge H, Twedt D.C, Ohta H, Nuruki T, Steiner J.M (2024). Management of acute-onset pancreatitis in dogs:A Narrative Review. J. Am. Vet. Med. Assoc.

[5] Guglielmini C, Crisi P.E, Tardo A.M, Di Maggio R, Contiero B, Boari A, Fracassi F, Miglio A (2022). Prognostic role of red cell distribution width and other routine clinico-pathological parameters in dogs with acute pancreatitis. Animals (*Basel*).

[6] Gori E, Lippi I, Guidi G, Perondi F, Pierini A, Marchetti V (2019). Acute pancreatitis and acute kidney injury in dogs. Vet. J.

[7] Cridge H, Langlois D.K, Steiner J.M, Sanders R.A (2023). Cardiovascular abnormalities in dogs with acute pancreatitis. J. Vet. Intern Med.

[8] Kuzi S, Mazor R, Segev G, Nivy R, Mazaki-Tovi M, Chen H, Rimer D, Duneyevitz A, Yas E, Lavy E, Aroch I.A (2020). Prognostic markers and assessment of a previously published clinical severity index in 109 hospitalised dogs with acute presentation of pancreatitis. Vet. Rec.

[9] Rimer D, Chen H, Bar-Nathan M, Segev G (2022). Acute kidney injury in dogs:Etiology, clinical and clinicopathologic findings, prognostic markers, and outcome. J. Vet. Intern Med.

[10] Keene B.W, Atkins C.E, Bonagura J.D, Fox P.R, Häggström J, Fuentes V.L, Oyama M.A, Rush J.E, Stepien R, Uechi M (2019). ACVIM consensus guidelines for the diagnosis and treatment of myxomatous mitral valve disease in dogs. J. Vet. Intern Med.

[11] Bugbee A, Rucinsky R, Cazabon S, Kvitko-White H, Lathan P, Nichelason A, Rudolph L (2023). 2023 AAHA selected endocrinopathies of dogs and cats guidelines. J. Am. Anim. Hosp. Assoc.

[12] Miyakawa H, Ogawa M, Sakatani A, Akabane R, Miyagawa Y, Takemura N (2021). Evaluation of the progression of non-azotemic proteinuric chronic kidney disease in dogs. Res. Vet. Sci.

[13] Mansfield C, Beths T (2015). Management of acute pancreatitis in dogs:A critical appraisal with focus on feeding and analgesia. J. Small Anim. Pract.

[14] Yamamoto S, Ohta Y, Hasegawa E, Hashida S, Kaneko Y, Mizutani S, Ong B.H.E, Naganobu K, Torisu S (2019). Usefulness of urinary creatinine/urea nitrogen ratio as indicator of body protein catabolism in dogs fed low protein diets. Front. Vet. Sci.

[15] Prause L.C, Grauer G.F (1998). Association of gastrointestinal hemorrhage with increased blood urea nitrogen and BUN/creatinine ratio in dogs:A literature review and retrospective study. Vet. Clin. Pathol.

[16] Webster C.R.L, Center S.A, Cullen J.M, Penninck D.G, Richter K.P, Twedt D.C, Watson P.J (2019). ACVIM consensus statement on the diagnosis and treatment of chronic hepatitis in dog. J. Vet. Intern Med.

[17] McKenna M, Pelligand L, Elliott J, Walker D, Jepson R (2020). Clinical utility of estimation of glomerular filtration rate in dogs. J. Vet. Intern Med.

[18] Yi K.C, Heseltine K.C, Jeffery N.D, Cook A.K, Nabity M.B (2023). Effect of withholding food versus feeding on creatinine, symmetric dimethylarginine, cholesterol, triglycerides, and other biochemical analytes in 100 healthy dogs. J. Vet. Intern Med.

[19] Braun J.P, Lefebvre H.P, Watson A.D (2003). Creatinine in the dog:A review. Vet. Clin. Pathol.

[20] Hall J.A, Yerramilli M, Obare E, Yerramilli M, Melendez L.D, Jewell D.E (2015). Relationship between lean body mass and serum renal biomarkers in healthy dogs. J. Vet. Intern Med.

[21] Uchino S, Bellomo R, Goldsmith D (2012). The meaning of the blood urea nitrogen/creatinine ratio in acute kidney injury. Clin. Kidney J.

[22] Stiller J, Defarges A.M, Brisson B.A, Bersenas A.M.E, Pomrantz J.S, Lang B, Pearl B.L (2021). Diagnostic evaluation of urea nitrogen/creatinine ratio in dogs with gastrointestinal bleeding. J. Vet. Intern. Med.

[23] Chikazawa S, Dunning M.D (2016). A review of anaemia of inflammatory disease in dogs and cats. J. Small Anim. Pract.

[24] Gianesini G, Drigo M, Zoia A (2023). Immune-mediated hemolytic anemia and clinically suspected acute pancreatitis in dogs, a pilot study. Top Companion Anim. Med.

[25] Segev G, Cortellini S, Foster J.D, Francey T, Langston C, Londoño L, Schweighauser A, Jepson R.E (2024). International Renal Interest Society best practice consensus guidelines for the diagnosis and management of acute kidney injury in cats and dogs. Vet. J.

[26] Chen H, Klainbart S, Kelmer E, Segev G (2022). Continuous renal replacement therapy is a safe and effective modality for the initial management of dogs with acute kidney injury. J. Am. Vet. Med. Assoc.

[27] Han S.M (2024). Predictive role of lactate in dogs with acute pancreatitis advanced to systemic inflammatory response syndrome. Vet. Res. Forum.

[28] Johnson M.M, Gicking J.C, Keys D.A (2023). Evaluation of red blood cell distribution width, neutrophil-to-lymphocyte ratio, and other hematologic parameters in canine acute pancreatitis. J. Vet. Emerg. Crit. Care (*San Antonio*).

[29] Keany K.M, Fosgate G.T, Perry S.M, Stroup S.T, Steiner J.M (2021). Serum concentrations of canine pancreatic lipase immunoreactivity and C-reactive protein for monitoring disease progression in dogs with acute pancreatitis. J. Vet. Intern Med.

[30] Araki R, Iwanaga K, Ueda K, Isaka M (2021). Intestinal complication with myxomatous mitral valve diseases in chihuahuas. Front. Vet. Sci.

[31] Mattin M.J, Boswood A, Church D.B, López-Alvarez J, McGreevy P.D, O'Neill D.G, Thomson P.G, Brodbelt D.C (2015). Prevalence of and risk factors for degenerative mitral valve disease in dogs attending primary-care veterinary practices in England. J. Vet. Intern Med.

